# Clonal Hematopoiesis in Cardiovascular Risk: Focus on Inflammatory Mechanisms

**DOI:** 10.3390/jcm15062393

**Published:** 2026-03-20

**Authors:** Siamala Sinnadurai, Michael C. Honigberg, Wouter C. Meijers, Daphne Merkus, Abhishek Niroula, Hari S. Sharma, Piotr Jankowski, Peter J. Van Der Spek, Rudolf A. de Boer, Olivier C. Manintveld, Karol A. Kaminski

**Affiliations:** 1 Department of Cardiology, Thorax Center, Cardiovascular Institute, Erasmus MC, 3015 GD Rotterdam, The Netherlandsr.a.deboer@erasmusmc.nl (R.A.d.B.); o.manintveld@erasmusmc.nl (O.C.M.); 2 Cardiology Division, Massachusetts General Hospital, Boston, MA 02114, USA; mhonigberg@mgh.harvard.edu; 3 Program in Medical and Population Genetics, Broad Institute of MIT and Harvard, Cambridge, MA 02142, USA; 4 Division of Experimental Cardiology, Department of Cardiology, Erasmus MC, 3015 GD Rotterdam, The Netherlands; d.merkus@erasmusmc.nl; 5 Department of Medical Biochemistry and Cell Biology, Institute of Biomedicine, Science for Life Laboratory, University of Gothenburg, 40530 Gothenburg, Sweden; abhishek.niroula@gu.se; 6 Cancer Program, Broad Institute of MIT and Harvard, Cambridge, MA 02142, USA; 7 Department of Pathology & Clinical Bioinformatics, Erasmus MC, 3015 GD Rotterdam, The Netherlands; h.sharma@erasmusmc.nl (H.S.S.); p.vanderspek@erasmusmc.nl (P.J.V.D.S.); 8 Department of Internal Medicine and Geriatric Cardiology, Centre of Postgraduate Medical Education, 01-813 Warsaw, Poland; piotr.jankowski@cmkp.edu.pl; 9 Department of Population Medicine and Lifestyle Diseases Prevention, Medical University of Bialystok, 15-089 Bialystok, Poland; karol.kaminski@umb.edu.pl

**Keywords:** clonal hematopoiesis, cardiovascular disease, inflammation

## Abstract

Clonal hematopoiesis (CH) is the expansion of clones from a single hematopoietic stem cell (HSC) in the bone marrow. Clonal hematopoiesis of indeterminate potential (CHIP) refers to CH defined by the presence of pre-leukemic driver mutations in at least 2% of alleles in sequenced peripheral blood. This phenomenon is, by definition, associated not only with the future development of acute myeloid leukemia but also with non-malignant conditions, including cardiovascular disease. However, the underlying molecular mechanisms for CH in non-malignant diseases, such as cardiovascular disease, are not fully explained. Certain subtypes of CHIP may give rise to proinflammatory immune cells, which, in turn, may promote atherosclerosis progression. Key subtypes of CHIP include mutations in genes encoding epigenetic regulators DNMT3A (DNA methyltransferase 3A), TET2 (ten-eleven translocation methylcytosine dioxygenase 2), and ASXL1 (associated sex combs-like 1), as well as mutations in the gene encoding hematopoietic cytokine signaling: JAK2 (Janus kinase 2). The aim of this review is to summarize the current knowledge of CHIP and its association with inflammation and cardiovascular risk factors.

## 1. Introduction

Somatic mutations in hematopoietic stem cells (HSCs) accumulate with age, particularly in cancer-related genes, predisposing these cells to clonal expansion [1,2,3,4]. These somatic mutations typically originate from leukemia-associated genes, affecting blood cells in individuals who do not exhibit any known hematologic malignancy, cytopenia, dysplasia, or other recognized inflammatory diseases. This condition, termed clonal hematopoiesis of indeterminate potential (CHIP), is characterized by the presence of a single driver somatic mutation with a variant allele frequency (VAF) of at least 2% in peripheral blood mononuclear cells [5,6].

CHIP is associated with an approximately 30–40% increase in overall mortality, primarily due to cardiovascular-related incidents and deaths [7]. Particularly, CHIP is linked to a twofold increase in the incidence of coronary artery disease and a fourfold increase in early-onset myocardial infarction compared to the risk of diagnosis with hematologic malignancies [6]. Given the growing body of evidence that supports the notion of CHIP as a novel and prevalent risk factor for cardiovascular disease, further investigation into its molecular and underlying mechanisms is warranted [6,8,9,10,11,12].

While immune system dysregulation has long been recognized as a key contributor to CVD, emerging evidence suggests that CHIP further exacerbates this mechanism through pathways of immunosenescence and inflammation. Immune markers of both adaptive and innate immunity are often altered with aging. These modifications lead healthy hematopoietic stem cells to acquire somatic mutations, which also give rise to mutated immune effector cells, such as monocytes, lymphocytes, and granulocytes [13]. For example, T lymphocytes undergo significant age-related changes, including a decline in diversity, enlargement of specific clones, and shifts in phenotypic characteristics [14,15]. These effector cells can potentially influence disease states, especially those with a chronic inflammatory component. Therefore, immune dysregulation is marked by elevated levels of circulating proinflammatory mediators and associated signaling pathways [16]. These mediators impair vascular function by altering endothelial cells, reducing the regenerative capacity of vascular progenitor cells, and promoting the proliferation of vascular smooth muscle cells. As a result, these changes may contribute to the transformation of atherosclerotic plaques from stable to vulnerable plaques [17,18,19], while elevating the risk of major cardiac events, including stroke, heart failure, and mortality [19,20].

Understanding the mechanisms of CHIP and cardiovascular disease (CVD) progression, particularly in the context of how somatic mutations contribute to immune dysregulation, is important for advancing our knowledge of disease pathogenesis. These mutations involve genes encoding epigenetic regulators, such as *DNMT3A* (DNA methyl transferase 3A), *TET2* (ten-eleven translocation methyl cytosine dioxygenase 2), *ASXL1* (associated sex combs-like 1), hematopoietic cytokine signaling JAK2 (Janus Kinase 2), DNA damage repair gene PPM1D (protein phosphatase, Mg^2+^/Mn^2+^ dependent 1D) and TP53 (tumor protein p53) as well as messenger RNA splicing factors SF3B1 (splicing factor 3b subunit 1) and SRSF2 (serine- and arginine-rich splicing factor 2), which reflect signs of potential hematological malignancy [20,21]. A key question is whether clonal hematopoiesis initiates cardiovascular complications, or if cardiovascular risk factors themselves act to fuel the expansion of clonal hematopoiesis, underscoring the complexity of this bidirectional relationship. Therefore, this review aims to address shared inflammatory mechanisms underlying the interplay between CHIP and CVD risk factors.

## 2. Prevalence of Chip: How to Detect?

Notably, age is a major determinant of CHIP prevalence, reflected by an increased frequency of detectable hematopoietic clones with advancing age. Older individuals are more likely to harbor CHIP clones compared to younger individuals, primarily due to prolonged exposure to various environmental factors that promote the accumulation of somatic mutations. These mutations facilitate the proliferation, expansion and maturation of clones, thereby enhancing their detectability with high-sensitivity sequencing technologies. Furthermore, the prevalence of CHIP is relatively higher in individuals with diseases such as CVD or hematologic malignancies compared to healthy populations [6,20]. For instance, whole-exome sequencing (WES) has revealed that 10–20% of individuals aged 70 or older harbor detectable CHIP clones [5,22], whereas fewer than 1% of individuals under the age of 40 exhibit detectable clones when analyzed using next-generation sequencing (NGS) techniques. From epidemiological studies using WES data, CHIP is highly prevalent in aging individuals without hematological phenotypes, with more than 10% of CHIP mutations identified in individuals aged > 70 years [22,23], followed by ~6% in those aged 60–69 years, ~12% in those aged 70–89 years, and ~20% in those aged ≥ 90 years [20,24]. Technically, CHIP clones are universally detectable in individuals over the age of 50 with deeper sequencing techniques. However, the clinical implications of very small clones (with a VAF of less than 1%) are less clear and require further experimental validation [24,25]. Importantly, depth sequencing technologies offer detection of CHIP somatic variants at even greater depths. Error-corrected sequencing techniques allow the detection of clonal events at levels as low as 0.03% VAF [26]. Therefore, the depth of sequencing and the age of the study population are crucial factors for accurate CHIP detection.

Although the accumulation of somatic mutations relative to age [27,28], progress in understanding this relationship has been hindered by the lack of technologies capable of reliably quantifying the distribution of somatic mutations across cells. The development of cutting-edge techniques, such as NGS, plays an important role in overcoming these limitations. Particularly, detecting somatic mutation genes (for example, *DNMT3A*, *TET2*, *ASXL1*, *JAK2*, *TP53*, *PPM1D*) through NGS involves calculating the VAF. If sequencing of the DNA sample shows five mutant reads and 95 wild-type reads, the VAF is calculated to be 0.05, meaning > 5% of the sequenced alleles possess the mutation. In the case of a heterozygous mutation in DNA, this corresponds to ~10% of cells carrying the somatic mutations or variant. CHIP variants are typically recognized at a VAF of 2% and above [6,9,29]. Whole-genome sequencing (WGS) and WES are the gold standard approaches for the detection of CHIP with VAF > 5%, but they may not be optimal for detecting CHIP mutant clones with VAF < 5%. More targeted approaches are required to detect mutations in the VAF range of 2–5%, with high-sensitivity error-corrected (e.g., using duplex sequencing) or deep targeted sequencing at equal 1650 coverage enabling the detection of clones with a VAF below 0.1% [9,24,29]. Some advanced methods, such as single-cell technologies, provide an abundance of information to aid the identification of CHIP with very small VAF. While somatic mutations with low VAF in a set of CHIP genes are significantly associated with mortality in chronic heart failure, the biological significance of these low VAF mutations remains unclear [22].

## 3. Mechanisms Underlying CHIP and CVD

### 3.1. Epidemiological Studies Looking at CHIP and CVD

Epidemiological cohort studies analyzing primary and secondary sequencing data provide strong evidence supporting the urge to investigate the relationship between clonal expansion frequency and CVD manifestation, particularly through the application of various NGS techniques (Supplementary File Table S1). CHIP has been associated with vascular diseases other than atherosclerosis. There are reports suggesting that individuals with CHIP or with mutations in CHIP driver genes (e.g., *TET2*) may have an increased risk of venous thromboembolic disease. This phenomenon, however, requires further prospective studies. These studies indicate that the clinical consequences of CHIP mutations strongly influence the dynamics of CHIP clones. Mutations involving established driver genes (e.g., *DNMT3A*, *TET2*, *ASXL1*, *PPM1D* and *TP53*) or passenger mutations (e.g., other AML-associated mutations) markedly alter pathogenic potential, consistent with classifications outlined by the American College of Medical Genetics and Genomics (ACMG) molecular pathology guidelines. The physical characteristics of clones, such as relative size and the rate of mutational expansion, further contribute to disease progression [6]. With that, evidence from large-scale datasets provides a powerful framework for exploring gene-environment interactions and offers valuable insights into the activation of inflammatory pathways relevant to both CHIP and CVD.

### 3.2. Animal Studies into Chip and Atherosclerosis

**TET2**: Jaiswal et al. [6] conducted studies in TET2-deficient mice to investigate the causal relationship between CHIP and atherosclerosis. Their research demonstrated that chimeric mice with Tet2 loss-of-function in hematopoietic stem cells in bone marrow developed significantly larger atherosclerotic plaques in the descending aorta after 17 weeks on a high-fat diet compared to mice transplanted with wild-type bone marrow. This effect occurred despite similar blood counts and serum cholesterol levels between the groups [30]. Similarly, Fuster et al. utilized a competitive bone marrow transplantation (BMT) approach to create atherosclerosis-prone LDLR-deficient mice carrying an initial 10% of Tet2-/- cells, mimicking the size of the human bone marrow niche. Both studies demonstrated that Tet2-/- cells expanded in the bone marrow, spleen and blood, affecting both myeloid and T-lymphoid lineages, which aligned with human population studies. In the mouse models, atherosclerotic plaques in the aortic root were approximately 60% larger compared to their wild-type counterparts [6,31]. Mechanistically, *TET2* deficiency activated the NLRP3 inflammasome in murine hematopoietic cells, leading to increased expression of inflammatory cytokines, including IL-6 and IL-1β [31]. Additionally, higher levels of chemokines such as Cxcl1, Cxcl2, and Cxcl3 were observed in TET2-deficient hematopoietic stem cells, indicating alterations in pathways specific to monocyte and T-cell subpopulations. These changes contribute to the increased risk of atherosclerotic cardiovascular disease (CVD) in CHIP carriers [10,31,32].

**DNMT3A**: Rauch et al. [23] investigated the effect of Dnmt3a loss-of-function in atherosclerosis. The model was created by transplanting bone marrow from mice with Dnmt3a loss-of- function in hematopoietic cells (knockout (KO): Dnmt3aafl/fl;Vav1-Cre) or control mice (wild-type (WT): Dnmt3+/+;Vav1-Cre) into irradiated atherosclerosis-prone Ldlr-/- mice. The recipient mice carried KO bone marrow, resembling the CHIP clone size observed in humans with Dnmt3a mutations. An atherogenic diet was given during hematopoietic reconstitution to promote atherosclerosis development. Mice transplanted with 10% knockout (KO) bone marrow developed 40% larger aortic root lesions compared to those with wild-type (WT) marrow after 9 weeks on a high-fat, high-cholesterol diet, suggesting that Dnmt3a loss-of-function accelerates atherosclerosis. To evaluate the contribution of Dnmt3a mutations to specific cell lineages in atherosclerosis, mice with a myeloid-specific deletion of Dnmt3a were generated by crossing Lyz2-Cre mice with Dnmt3a floxed allele mice. Bone marrow from these Dnmt3afl/fl;Lyz2-Cre mice was then transplanted into atherosclerosis-prone Ldlr-/- mice. Compared to wild-type, these mice showed 46% larger aortic root lesions and increased content of lesional macrophages, indicating that Dnmt3a mutation loss in myeloid cells significantly accelerates atherosclerosis. Loss of DNMT3A enhanced inflammation in macrophages in vitro and generated a distinct macrophage population in vivo that merges a resident macrophage profile with an inflammatory cytokine signature. The macrophage mutant cell population expanded between the 24- and 30-week time point is highly chemotactic, and its inflammatory signature implicates it as a putative cellular source of the chemokines that recruit myeloid cells to inflamed vessel walls. Among the most highly enriched processes were cytokine–cytokine receptor interaction, tumor necrosis factor (TNF) signaling, interleukin 17 signaling and atherosclerosis-related pathways. The results identify a common pathway promoting heightened innate immune cell activation, with the loss of either gene providing a biological basis for the excess atherosclerotic disease burden [23].

**TP53**: Zekavat et al. [33] investigated the impact of DNA damage response genes by using p53-deficient mice as a surrogate for human *TP53* mutations. To examine whether p53-deficient hematopoietic stem cells contribute to atherosclerosis, they created chimeric Ldlr-/- mice with 20% Trp53-/- hematopoietic cells. This chimerism is consistent with the VAF of *TP53* mutations identified in human studies (mean VAF = 0.13 i.e., ~26% mutant cells if monoallelic mutations; mean VAF = 0.23 in those defined as carrying large CHIP clones). At 13 weeks post-grafting, these mice showed a ~40% increase in aortic plaque size and enhanced macrophage accumulation, without changes in body weight or cholesterol levels. These findings suggest that p53-deficient macrophages in the murine atherosclerotic aorta increased atherogenesis by selectively expanding within plaques, contributing to accelerated atherosclerosis in p53-deficient CHIP conditions [33].

**JAK2**: Fidler et al. [34] studied the impact of the JAK2 V617F mutation, which enhances JAK-STAT signaling, in the progression of atherosclerotic cardiovascular disease. To assess the clonal expansion of bone marrow-derived *JAK2* macrophages within atherosclerotic lesions, lethally irradiated Ldlr-/- mice were transplanted with bone marrow cells expressing Mx1-cre Jak2VF Confetti or Mx1-cre Confetti. Three weeks post-transplantation, Cre-recombinase was induced using two intraperitoneal injections of pIpC (200 µg/mouse/day), followed by 16 weeks on a high-fat diet. After euthanasia, the murine hearts and aortas were perfused and frozen for further measurements. RNA sequencing on myeloid cells from wild-type mice transplanted with Mx1-cre Jak2VF showed that the expression of Jak2VF was associated with enrichment for genes involved in cellular proliferation, DNA damage repair, and metabolic pathways. Recruitment of macrophages (derived from blood monocytes) to injury sites, aiding plaque formation, is a key process in atherosclerosis. This was further studied using tamoxifen-treated Ldlr-/- transgenic mice on a 15-week Western diet, transplanted with bone marrow from Cx3cr1-creJak2VF mice, which showed increased lesion areas, macrophage proliferation, and necrotic core formation. These results suggest that Jak2VF expression in macrophages drives their proliferation and promotes necrotic core formation. Additionally, increased IL-1β expression in lesion macrophages indicated enhanced inflammasome activation. In further testing, Mx1-cre Jak2VF mice crossed with Casp1/11-/- mice showed elevated serum levels of IL-18, an inflammasome product, and decreased cholesterol levels, consistent with findings in human studies that CHIP-related mutations drive inflammation [35,36].

### 3.3. Inflammation and CHIP in CVD

Inflammation is defined as a cellular and humoral response to injury [37]. Immune markers of inflammation, including leukocytes (e.g., monocytes, macrophages, neutrophils, lymphocytes), serum proteins (e.g., C-reactive protein), and cytokines (e.g., IL-6), play significant roles in this process [38]. In the context of CHIP, the presence of somatic mutations is often associated with increased inflammation via cytokine regulation; however, the causal relationship between mutations and specific inflammatory mediators has not yet been fully established [39]. Cytokines, particularly the upregulation of IL-1, contribute to the pathogenesis and progression of proinflammatory conditions, thereby promoting increased CVD risk and severity [40,41]. Initial results have shown that *DNMT3A* and *TET2* mutations lead to chronic inflammation in the tissue microenvironment of CVD development and progression [10,42]. This signal was detected in the elevation of c-reactive protein, an acute response protein synthesized in the liver in response to various inflammatory stimuli, a known validated residual inflammatory marker in CVD [43,44]. Hematopoietic stem cells (HSCs) carrying these mutations are more often present in circulating leukocytes and exhibit enhanced monocyte recruitment activity. Furthermore, each of these mutations serves as a major contributor to the three principal hallmarks of atherosclerosis: excessive lipid accumulation, vascular smooth muscle proliferation, and immune cell infiltration within the vascular wall [45].

Dysregulation of cytokines plays an important role in atherosclerosis by promoting monocyte recruitment, adhesion, transmigration through the endothelium, and macrophage transformation [46,47]. Of these, the most prominent cytokines are CXC family chemokines, IL-6, and IL-1β, which are upregulated due to CHIP mutations [6,31]. It has been speculated that an injured vascular endothelium tends to recruit or be more susceptible to monocytes carrying *DNMT3A* and *TET2* mutations or other activated immune-competent cells to the respective injured areas, causing local inflammation. This proinflammatory environment is partly driven by inflammasome activation, a key component of the innate immune response, which induces pyroptotic cell death and promotes the secretion of IL-1β and IL-18 through caspase-1 activation and release from the inflammasome complex. Specifically, a mutation in the *DNMT3A* leads to monocyte activity via cytokine expression. The activated monocytes communicate with non-mutated monocytes by amplifying cytokine regulation [10]. Mutant monocytes also stimulate other immune cells, like T-cells, to become more inflammatory, even without the *DNMT3A* mutation. This complex interplay contributes to an inflammatory environment in the circulatory system of CVD patients [48], which was later also shown in larger population studies [35]. In population studies incorporating single-cell transcriptomic technologies, CXC chemokines and IL-8 and its receptors trigger monocyte adhesion to vascular endothelium [10]. Particularly, in the transcription analysis assessing these leukocytes, the underlying mechanism suggests activated proinflammatory markers, such as IL-6, IL-1β, TNF-α and IFN-γ, and involves the assembly known as the NLRP3 inflammasome. Inflammasome activation was stimulated by genetic features of *DNMT3A* and *TET2* mutation, with loss of function eventually activating the NLRP3 inflammasome, while the *JAK2VF* mutation, with gain of function, activates the AIM2 inflammasome [6,12,31,34].

Recently, advances in high-throughput plasma protein measurements have allowed for the establishment of protein risk profiles that outperform clinical risk markers in predicting cardiovascular prognostic markers [49]. Moreover, integrating proteomics with the genetic “proteogenomics” signatures of CHIP mutations has revealed an abundance of novel biomarkers, including proteins that are implicated in cytokine signaling in immune system regulation of IL-1β/NLRP3/IL-6 pathways, such as proteins belonging to TNFRSF (TNFRSF9 and TNFSF14) [50] and FLT3 levels [51,52]. This inflammatory profiles revealed the dysregulation of cytokines and interleukins as crucial in major adverse cardiac events in patients with stable coronary artery disease, even with controlled LDL cholesterol, as demonstrated in the CANTOS trial [53]. Genetic expression present during the progression of atherosclerosis also suggests their potential involvement in the inflammatory processes contributing to atherosclerotic plaque formation. Observational studies have reported elevated levels of cytokines such as CRP in patients with stable coronary artery disease (CAD), indicating a potential role for inflammation that may be driven by clonal hematopoiesis [54]. Additionally, specific somatic mutations in driver genes such as *DNMT3A* or *TET2* in patients with ST-segment elevation myocardial infarction (STEMI), with higher levels of the inflammatory cytokines IL-1β and IL-6, were associated with worse outcomes [55].

## 4. NLRP3 Inflammasome Activation in CHIP Contributes to NLRP3 Inflammasomes Activation

**Figure 1:**
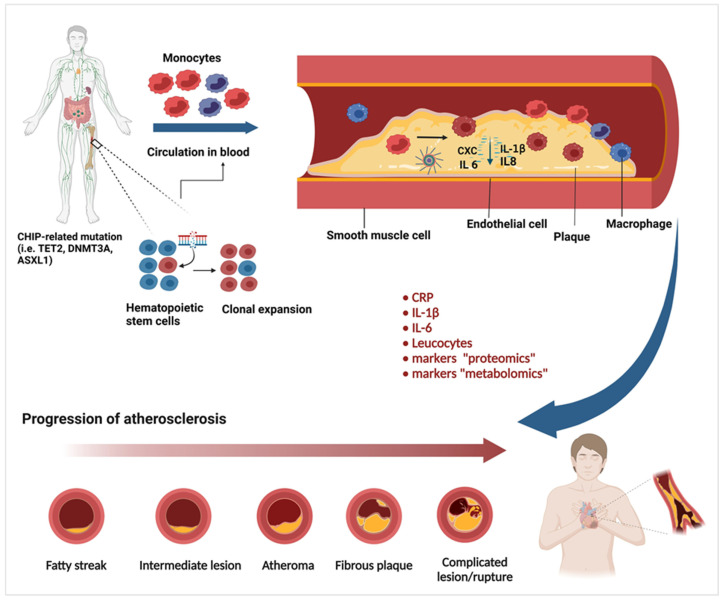
An illustration of how TET2 mutations may be associated with atherosclerosis. (A) HSCs in the bone marrow acquire somatic mutations in the *TET2* gene, TET2-deficient (red) and wild-type (blue). (B) Due to the selective advantage acquired by HSCs with TET2 mutations, the proliferation of mutant HSCs and HSC-derived monocytes (TET2-deficient) leads to greater growth of the mutant cells than of wild-type cells in the bone marrow and blood. (C) In the arteries, monocytes, especially (TET2-deficient) monocytes, are recruited to plaques, where they give rise to inflammatory macrophages. A complex molecule called the inflammasome in macrophages cleaves and activates the protein IL-1β, which is secreted and induces the expression of the protein P-selectin in endothelial cells that line blood vessels. (D) This increases the recruitment of monocytes to the plaques, which leads to more macrophages, causing further inflammation that accelerates the progression of atherosclerosis. (E) This mechanism is similar to the known progression mechanism of atherosclerosis, leading to the manifestation of MACE (i.e., myocardial infarction, stroke and heart failure).

A summary of the aforementioned mouse studies and knowledge of inflammation in CVD demonstrates that *TET2* and *DNMT3A* somatic mutations causally induce immune cell proliferation and promote inflammation through the activation of multiple inflammasomes, the most prominent of which is the NLRP3 inflammasome. In general, NLRP3 activation follows a two-step process: priming and activation. The priming stage is initiated by the engagement of Toll-like receptors (TLRs) that recognize pathogen-associated molecular patterns (PAMPs) or danger-associated molecular patterns (DAMPs), subsequently activating nuclear factor kappa B (NF- kB) signaling. This transcriptional cascade induces the expression of NLRP3 and pro-IL-1β, thereby establishing a sensitized state for subsequent inflammasome activation. Structurally, the NLRP3 inflammasome comprises three essential domains: the nucleotide-binding and oligomerization (NACHT) domain, the leucine-rich repeat (LRR) domain, and the pyrin domain (PYD), which together orchestrate oligomerization and downstream inflammatory signaling [56]. Once the NLRP3 domains assemble, this form triggers the activation of caspase-1, which proteolytically activates the proinflammatory cytokine IL-1β [57]. To date, it remains unclear how both *TET2* and *DNMT3A* mutations contribute to the assembly and activation of the NLRP3 inflammasome complex. A murine model of CHIP-mediated atherosclerosis revealed that stimulation of Toll-like receptors (TLRs), the IL-1 receptor (IL-1R), or tumor necrosis factor receptor (TNFR) in macrophages through the canonical pathway led to the activation of NLRP3 (Figure 1).

Clonal populations arising from mutated monocytes, particularly those harboring *TET2* or *DNMT3A* mutations, contribute to enhanced recruitment and retention of myeloid cells at sites of vascular injury, thereby promoting inflammatory cell accumulation and the build-up of apoptotic and necrotic cells within the lesion microenvironment. Consistently, Sano et al. demonstrated that Tet2-deficient myeloid cells drive IL-1β–mediated inflammation and adverse cardiac remodeling, supporting a pathogenic role for mutant myeloid cells in cardiovascular injury [32]. These subgroups of cells release signaling molecules that propagate inflammatory crosstalk with adjacent macrophages, triggering pyroptotic cell death and further amplifying local vascular inflammation.

**Figure 2:**
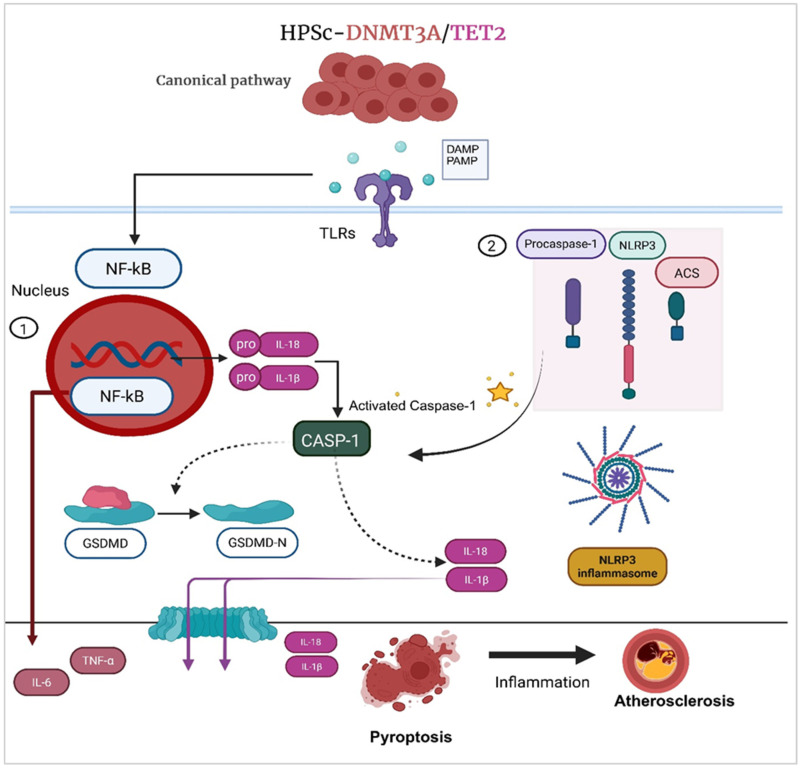
Proposed mechanism by which mutant HSCs promote pyroptosis and inflammation in cardiovascular disease. *TET2* and *DNMT3A* give rise to clonal populations of myeloid cells with a proinflammatory phenotype. Mutant monocytes and their derived macrophages are preferentially recruited to sites of vascular injury, where they exhibit heightened activation of inflammasome pathways (e.g., NLRP3). (1) NF-ĸb is a transcription factor activated in response to pathogen signals. In mutant monocytes/macrophages, epigenetic changes enhance NF-ĸb activation, allowing it to translocate into the nucleus and induce transcription of pro-IL-1β and pro-IL-18. (2) Inflammasome assembly leads to caspase-1 activation, cleavage of gasdermin D, and induction of pyroptotic cell death, accompanied by the maturation and release of interleukin-1β (IL-1β) and interleukin-18 (IL-18). The resulting amplification of local inflammation promotes further immune cell recruitment, accumulation of apoptotic and necrotic debris, and progression of atherosclerotic lesions.

Pyroptosis is a mechanism of programmed cell death that contributes to inflammation and tissue damage. It involves a sequence of molecular events, including inflammasome activation, caspase-1 cleavage, gasdermin D pore formation and release of the proinflammatory cytokines IL-1β, IL-6, TNF-α, IL-18, and MCP-1 [58]. Current understanding of inflammasome signaling identifies caspase-1-mediated pyroptosis as a critical innate immune effector mechanism [48]. Pyroptosis not only drives local inflammation but also amplifies the systemic inflammatory response, thereby exacerbating atherosclerotic plaque instability. This progression eventually leads to plaque rupture, thrombosis, and major adverse cardiac events [59]. Notably, this mechanism may explain the relationship between CHIP and atherosclerosis, particularly in the context of specific gene mutations that contribute to the elevation of proinflammatory cytokines, consequently activating downstream inflammatory pathways (Figure 2).

## 5. CVD Risk Factors Linked to CHIP

**Cardiometabolic risk factors**

Elevated insulin level and type 2 diabetes as well as increased body weight and obesity

Common cardiometabolic factors, such as obesity, insulin resistance and high blood cholesterol, promote a proinflammatory state, potentially playing a pivotal role in driving CHIP-associated biomarkers. A study involving individuals with obesity (n = 273) and CHIP revealed that nearly 80% of obese individuals displayed clones with VAF ranging from 0.01% to 31.2%. Subsequently, in a follow-up analysis spanning 20 years, 18.5% (n = 40) of CHIP-positive samples showed that small clones could evolve into CHIP over time, with VAF increasing by an average of 7% (range −4% to 27%) per year. Notably, the rate of clonal growth was significantly associated with insulin resistance (*p* = 0.025) and low circulating levels of high-density lipoprotein cholesterol (HDL-C) (*p* = 1.74 × 10^−5^). Further evidence showed that obesity can induce a phenotype that accelerates the expansion of pre-leukemic HSCPs carrying *DNMT3A* or *TET2* mutations, leading to a severe myeloproliferative neoplasms-like phenotype. A competitive transplantation assay in obese bone marrow recipient mice revealed a significant difference in pre-leukemic CHIP mutant HSCPs (CD45.2+ cells) supporting competitive advantage, clonal expansion, and subsequent malignancy. These mice also exhibited elevated fasting blood glucose levels and increased heart weights compared to controls. Moreover, the obesity phenotype was found to regulate Ca2+ levels in *TET2-/-* HSCs, suggesting a potential link between intracellular metabolism and epigenetic modifications [50]. Similarly, Fuster et al. demonstrated that increased insulin resistance under conditions of *TET2* loss of function in HSCs is primarily facilitated by exacerbated IL-1β expression in aged mice on a high-fat/high-sucrose (HF/HS) obesogenic diet [42]. Conversely, epidemiological data showed that a healthy lifestyle, especially having a normal body mass index, was strongly associated with a lower prevalence of CHIP, suggesting that controlling certain cardiometabolic factors through healthy lifestyle changes could reduce the incidence of CHIP [60]. Taken together, given the high prevalence of CHIP in CVD patients, it should be understood that the competitive selection of HSCPs depends on both the type of somatic mutations and cardiometabolic factors. This underscores the importance of addressing these factors in the context of CHIP-associated pathologies and potential therapeutic and preventive strategies for cardiovascular diseases.

**Cigarette smoking and excessive DNA damage**

Several studies have highlighted smoking as a significant factor associated with somatic mutations in nuclear DNA as well as mitochondrial DNA in multiple tissues, which has also been linked to CHIP in various investigations [61,62]. An observational study among participants in the UK Biobank revealed a strong association between smoking and CHIP, particularly through mosaic chromosomal changes. However, when the analysis was repeated using two-sample Mendelian randomization with adjustments for confounders, smoking still showed a robust correlation with extended large structural variants, but not with CHIP-related extended variant driver mutations. Nevertheless, it has been suggested that smoking may have a causal relationship with CHIP. Smoking is known to create toxic environment that can cause DNA damage and trigger leukocytosis, which may accelerate HSC proliferation and potentially influence the fitness of clones with *ASXL1* gene mutations [63,64]. Moreover, smoking has also been associated with other genomic changes, such as loss of the Y chromosome (LOY), mosaicism, and loss of *ASXL1* function [65]. While the acquisition and clonal expansion of mutations that alter stem cell fitness are well-established hallmarks of carcinogenesis, their role in cardiovascular disease remains unclear due to limited scientific literature on the subject. Further research is needed to elucidate the complex relationship between smoking, somatic mutations, and the development of cardiovascular disease.

**Stress and sleep disorders**

Chronic stress is a known risk factor for various diseases, including cardiovascular disease. Research by Heidt and colleagues has shown that psychosocial stress can promote CHIP by increasing hematopoietic stem cell activity. Stress leads to a higher rate of proliferation of HSCs, which in turn favors the production of inflammatory leukocytes that can contribute to disease progression. In a mechanistic model, exposing atherosclerosis-prone ApoE-/- mice to chronic stress resulted in accelerated CHIP, affecting plaque structures that are more susceptible to vulnerable lesions, leading to conditions like myocardial infarction and stroke [66]. In a comparable study, the impact of CHIP-associated mutations, particularly *TET2*, was investigated in the context of sleep deprivation. Ldlr-/- mice showed a significant 1.6-fold increase in the prevalence of immunophenotypic features of Tet2-/- clones with sleep fragmentation, confirming the emergence of CHIP [67]. These studies support the association of CHIP with severe chronic stress and sleep disturbance, potentially providing an additional pathway underlying the pathophysiology of atherosclerosis. Stress and sleep deprivation may have a bidirectional relationship, with each potentially modulating the other and collectively promoting adverse cardiovascular outcomes [68,69]. Altogether, these studies underscore the intricate interplay between stress, sleep, and clonal hematopoiesis in the development and progression of cardiovascular diseases.

**Role of gut microbiome**

The gut microbiome plays a critical role in influencing the overall health of the host and is implicated in the development of various diseases such as inflammatory bowel disease (IBD) and Crohn’s disease. Disruptions in the ‘microbial homeostasis’ within the gut are now recognized as a risk factor for leaky gut, atherosclerosis and other CVDs [70,71,72,73]. A leaking gut allows the gut microbiome to enter the bloodstream and cause infections, for example in the heart valves. Alterations in the composition of the gut microbiome can promote the competitive advantage of HSC with CHIP mutations, leading to clonal expansion. Moreover, the loss of *TET2* function in mice exposed to microbial signals, such as lipopolysaccharide (LPS), exacerbates a strong inflammatory response by triggering inflammatory genes that activate IL-6, suggesting that the interplay between CHIP mutations and microbial-induced environmental stressors contributes to clonal evolution [74]. Mechanistic studies have shown that microbial signals can influence the inflammatory signature of HSCs, and this effect may be reversible through circulating microbe-associated activity and increased production of IL-6 and IL-1 cytokines [74,75]. To date, the link between the gut microbiome and CHIP-related mutations has only been demonstrated in mouse models, so testing this hypothesis in humans could provide new insights into the association between specific microbiome-related traits and CHIP mutations and how this relates to atherosclerotic phenotypes. Understanding these complex relationships may pave the way for novel therapeutic approaches targeting the gut microbiome to mitigate the risk of CVDs associated with clonal hematopoiesis.

**Germline vs. Somatic mutation**

Emerging evidence indicates that germline variation can predispose individuals to CHIP by influencing somatic mutation acquisition and clonal expansion. Large-scale whole-genome sequencing studies have identified significant associations between germline variants in telomere-regulating genes, particularly TERT, and somatic CHIP mutations such as TET2 [12]. Although CHIP is commonly associated with age-related telomere shortening, Mendelian randomization analyses suggest a more complex relationship, whereby inherited alleles promoting telomere maintenance may increase susceptibility to CHIP by extending HSC replicative lifespan [76,77]. Similarly, germline variants in POT1, a critical regulator of telomere protection and telomerase activity, have been shown to delay cellular senescence and enhance clonal persistence [78]. These findings support a model in which inherited telomere biology shapes somatic mutation dynamics. In a familial VTE cohort, whole-genome sequencing of 216 individuals from 35 Han Chinese pedigrees identified 36 rare variants explaining 97% of families, including novel variants in GP6, TET2, and JAK2. Functional assays confirmed altered expression of GP6 and TET2. Given that TET2 and JAK2 are established CHIP drivers linked to inflammation and thrombosis, collectively, these findings suggest convergence between inherited susceptibility and clonal hematopoietic mechanisms in VTE and other CVD pathogenesis [79,80].

**Future directions**

Further research in CHIP should prioritize exploring potential gene-specific strategies that alter immune responses and inflammatory pathways to address CVD complication. In particular, focus on potential genetic and environmental factors that modulate CHIP risk. Therefore, exploring the mechanisms by which clonal hematopoiesis (particularly the rate of clonal expansion) contributes to the progression of atherosclerosis and major adverse CVD events is essential. In addition, the accumulation of somatic mutations in highly proliferative hematopoietic cells results in aberrant HSPC proliferative advantages, leading to clonal expansion. It is established that aging is associated with compromised hematopoietic and immune function. Notably, some individuals exhibit heightened clonal expansion in CHIP driver mutations with a larger VAF during midlife in the hematopoietic system. Among these CHIP carriers, factors determining who develops CVD are unclear [81]. A study reported that some mutant clones do not expand and remain relatively stagnant for more than 10 years or shrink before reverting to the end stage of the disease [24,82]. It is conceivable that these clones diminish in size in the presence of a more robust CHIP/non-CHIP clone [83]. Given this, the clonal expansion of individual CHIP genes within an individual is more likely to be random and linear [65]. This suggests that clonal expansion is triggered by variations in extrinsic and intrinsic genetic factors that alter the bone marrow niche and hematopoietic stem cells. Ultimately, the aim is to gain a comprehensive understanding of the complex interplay that drives clonal expansion within hematopoietic systems, which may influence the process or biological pathway of multi-clonal expansion.

**Conclusions and Perspectives**

CH is gaining attention across various disciplines, including cancer, CVD, and autoimmune conditions, with a specific focus on its implications in CVD. In the dynamic field of CH and CVD research, advancements in technology facilitate a deeper understanding of the biological aspects, holding transformative potential. Studying clonal dynamics at a single-cell level provides insight into the specific cellular subsets driving CH-related inflammation and immune responses within hematopoietic populations. This deepened biological knowledge is pivotal in identifying therapeutic targets and developing personalized interventions. Collaborative efforts among genomics, cardiology, and biology experts are essential to fully realize the potential of these biological insights, advancing more effective strategies for preventing and managing CVD associated with clonal hematopoiesis.

## Data Availability

No new data were created or analyzed in this study.

## Supplementary Materials

The following supporting information can be downloaded at https://www.mdpi.com/article/10.3390/jcm15062393/s1: Table S1: Results of primary and secondary cohort studies linked to CHIP and CVD; Table S2: CHIP driver genes linked to cardiovascular disease. References [84,85,86,87,88,89,90,91,92,93,94,95,96,97] are cited in Supplementary Materials.
