# Supplementary material for: Clonal Hematopoiesis in Cardiovascular Risk: Focus on Inflammatory Mechanisms
Source: J Clin Med. 2026 Mar 20;15(6):2393. doi: 10.3390/jcm15062393 (PMC13026250; doi:10.3390/jcm15062393)
Supplement: Supplementary file 1 [file jcm-15-02393-s001.zip › jcm-4143026-supplementary.pdf]

**Table S1. Results of primary and secondary cohort studies linked to CHIP and CVD.**

| Author , year | Study Objective | Study Population | Study outcome |
|---|---|---|---|
| Jaiswal, et al (2014) [20] | This study aims to investigate the prevalence and characteristics of somatic mutations that associated with hematologic malignancies in individuals without known hematologic disorders. | This study analysed whole-exome sequencing data from DNA in the peripheral-blood cells (pbmc) of 17,182 persons who were unselected for hematologic phenotypes. | The study concluded somatic mutations that drive clonal expansion of blood cells were a common finding in the elderly and most frequently involved *DNMT3A*, *TET2*, or *ASXL1*. Age-related (CH) is a common premalignant conditions that is also associated with increased overall mortality and increased risk of cardiometabolic disease. |
| Jaiswal, et al (2017) [6] | This study aims to investigate the association between clonal hematopoiesis of indeterminate potential (CHIP) and atherosclerotic cardiovascular disease (ASCVD). | This study analysed whole-exome sequencing data to detect the presence of CHIP in pbmc and associated with coronary heart disease using samples from our case-control studies that together enrolled 4726 participants with coronary heart disease and 3529 controls. | The study concluded the presence of CHIP in pbmc was associated with nearly a doubling in the risk of coronary heart disease in humans and with accelerated atherosclerosis in mice. |
| Dorsheimer L, et al. (2019) [9] | This study aimed to assess the potential prognostic significance of CHIP in patients with chronic heart failure (CHF) of ischemic origin. | This study analysed deep targeted amplicon sequencing to detect the presence of CHIP in bone marrow-derived mononuclear cells from 200 patients with CHF. | The study found that somatic mutations in hematopoietic cells, particularly in *TET2* and *DNMT3A*, were significantly associated with disease progression and poor prognosis of CHF. The author recommended validation of the findings in larger cohorts and suggest targeting specific inflammatory pathways may be valuable for precision medicine in patients with CHF carrying specific mutations encoding CHIP. |

| | | | |
|---|---|---|---|
| Bick AG, et al (2020) [12] | This study investigate the root cause of CH mutation (e.g., *DNMT3A* and *TET2*-CHIP) in germline and somatic whole genome sequence data to identify its association with CVD. | This study analysed high-coverage whole-genome sequences from 97,691 participants of diverse ancestries in the National Heart, Lung, and Blood institute Trans-omics for Precision Medicine (TOPMed) programme. | This study demonstrated that germline genetic variant influences the development of CH through gene-specific mechanisms. It was shown CHIP driver genes were associated with specific inflammatory traits. A *TET2* locus mutation enriched in individuals of African ancestry acts as a causal variant by disrupting a distal enhancer, resulting in increased self-renewal of HSCs. Its highlighted role of inherited genetic variation in shaping CH and somatic mutation acquisition. |
| Bick AG, et al. (2020) [34] | This study aim to investigate whether IL-6 pathway antagonism can reduce CVD risk in individuals with CHIP. | This study analysed whole exome sequencing data from 35, 416 individuals in the UK Biobank to identify CHIP carriers with *DNMT3A* or *TET2* mutations who have no prior history of CVD. | The results concluded that CHIP is associated with an increased risk of incident CVD. Among CHIP carriers with large clonal expansions, genetically reduced IL-6 signaling mitigated this risk. Specifically, the IL6R p.Asp358Ala variant was associated with a reduced incidence of CVD events in individuals with large CHIP clones, but not in those without CHIP. |
| Lambert Busque, et al. (2020) [52] | To investigate the relationship between inflammation and CHIP, with a focus on how inflammation may contribute to CHIP initiation and clonal expansion. | This study analysed targeted deep sequencing exome data focusing on 11 genes in 1887 subjects aged >70 years from the Montreal Heart Institute Biobank, of which 1359 had prior coronary artery disease (CAD), and 528 controls. | This study concluded, CHIP was identified in 22.6% of individuals, with *DNMT3A* and *TET2* being the most frequently mutated genes. *TET2* mutations were more common in controls than in CAD patients. CHIP carriers exhibited significantly elevated hs-CRP levels, supporting a link between CH and systemic inflammation. These findings support the hypothesis that inflammation may promote clonal expansion and suggest targeting inflammation therapeutic may attenuate CHIP-associated disease risk. |

| | | | |
|---|---|---|---|
| Pascual-Figal, et al (2021) [27] | This study assessed the clinical impact of CH on HF progression irrespective of its etiology. | This study analysed deep sequencing data of 62 patients with heart failure and left ventricular ejection fraction (LVEF) and identify CHIP mutations with a VAF >2%. Patients were prospectively followed for adverse cardiovascular outcomes over 3.5 years to assess the prognostic relevance of CHIP. | This study revealed somatic mutations that drive CH are common among HF patients with reduced LVEF and are associated with accelerated HF progression regardless of etiology. |
| Kar SP, et al (2022) [76] | This study investigate the cause and consequence of CH. | This study analysed whole-exome sequencing data from 200,453 UK Biobank participants. | This study concluded the number of germline variants associated with CH in individuals of European ancestry. Newly identified loci marker implicated biological pathways involved in CH development, including DNA repair (PARP1, ATM, CHEK2), stem cell function (CD164), and oncogenesis (SETBP1). Several associations were specific to CH subtypes, including variants at TCL1A and CD164, which demonstrated opposing effects in *DNMT3A*- versus *TET2*-mutant CH. Mendelian randomization analyses indicated that smoking and longer leukocyte telomere length are causal risk factors for CH, and that genetic predisposition to CH increases the risk of myeloproliferative neoplasia, nonhematologic malignancies, atrial fibrillation, and epigenetic aging. |
| Kessler MD, et al (2022) [84] | This study aims to investigate the role of CH in the expansion of specific blood cell lineages and its | This study analysed exome sequence data from 628,388 individuals from UK Biobank and identified 40,208 carriers of | This study demonstrated the relationship between CHIP and severe COVID-19 outcomes, cardiovascular disease, haematologic traits, malignancy, smoking, obesity, infection and all-cause mortality. CHIP was specifically linked to solid cancers, including non-melanoma skin |

| | | | |
|---|---|---|---|
| | association with aging and adverse health outcomes. | clonal haematopoiesis of indeterminate potential (CHIP). | cancer and lung cancer, while *DNMT3A*-related CHIP was associated with the development of myeloid but not lymphoid, leukaemias. |
| Nakao T, et al (2022) [77] | This study aims to investigate the bidirectional association between CHIP, leukocyte telomere length (LTL), and CAD. | This study analysed whole genome sequencing data from two large-scale cohorts UK Biobank (n = 47,080) and TOPMed (n = 63,,302) for observational studies, mandelian randomization for causal inferences , and mediation analyses. | This study demonstrated an inverse relationship between LTL and CHIP, with bidirectional Mendelian randomization suggesting that longer LTL promotes CHIP acquisition, while CHIP may contribute to LTL shortening. Although complex and partially counterintuitive, these findings align with prior observations in related hematologic phenotypes. Mediation analysis indicated that LTL shortening may modestly mediate the association between CHIP and CAD. |
| Yu Z, et al (2023) [85] | This study investigates the relationship between CHIP and CAD by analysing plasma proteomic profiles to identify potential biomarker and mechanistic pathways. | This study analysed whole genome sequencing data of 48,922 UK Biobank participants. | The results showed TET related CAD linked to extracellular matrix-related proteins,TIMP3, an ECM-bound protein inhibiting a broad range of substrates, including matrix metalloproteinase. These proteins involve in cardiac remodelling, cardiomyopathy and heart failure. While, *DNMT3A*-CAD linked to signaling and adhesion proteins in neural cells, contactin-1 involves in neural development and function. |
| Stacey SN, et al (2023) [86] | This study aimed to examine the genetic and epidemiological determinants of mutational barcode-defined CH, identifying key mutations, population-level associations, and potential disease outcomes. | This study analysed whole genome sequencing of 45,510 Icelandic and 130, 709 UK Biobank participants. | The results demonstrated that smoking has a dosage-dependent effect on the risk of CH. CH associates with several smoking-related diseases. However, there was no evidence that CH is associated with cardiovascular disease. |

| | | | |
|---|---|---|---|
| Yu Z, et al (2023) [87] | This study aimed to identify inflammatory gene modifiers related to the inflammasome pathway that influence CVD risk in individuals with CHIP. | This study analysed whole-exome sequencing data from 424,651 UK Biobank participants to identify CHIP. | The study identified IL1RAP and AIM2 as key modifiers of CHIP-associated CVD risk, with AIM2 expression enhancing CVD risk in *JAK2*- and *ASXL1*-mutant CHIP. Functional experiments showed that Asxl1-mutant macrophages had a heightened inflammatory and DNA damage response to AIM2 activation. Additionally, increased IL-10 expression may have a protective role in *ASXL*1-related CHIP, supporting the relevance of inflammasome pathways in CHIP-associated CVD. |
| Mayerhofer E, et al (2023) [88] | This study aimed to identify whether CHIP genetic screening can detect myeloid precursor lesions or covert myeloid neoplasms in young patients with stroke of undetermined etiology. | This study analyzed targeted sequencing data from patients with acute brain ischemia and compared CHIP prevalence to age-matched healthy controls from the Nijmegen Biomedical Study (n = 1,604) and UK Biobank (n = 101,678) | This study found a threefold higher CHIP prevalence in young patients with stroke of undetermined etiology compared to the general population. High-risk CHIP and covert myeloproliferative neoplasms were identified as potential stroke causes in a subset of patients, highlighting the diagnostic and therapeutic value of genetic screening. |
| Gumuser GD, et al (2023) [89] | This study aims to evaluate the impact of CHIP on adverse outcomes in patients with ASCVD. | This study analysed whole exome data from the UK Biobank cohort of patients establish ASCVD. | This study identified CHIP is independently associated with adverse outcomes in individuals with established ASCVD, with especially high risks observed in *TET2* and *SF3B1/SRSF2/U2AF1* CHIP. |
| Shi C, et al (2023) [90] | This study aimed to investigate the association of CHIP with incident HF in a European population cohort. | This study analysed error-corrected sequencing data from 705 participants, comprising individuals with incident HF and 1:1 age and sex matched control subjects, selected from | This study concluded that, individuals younger than 65 years, CHIP was more prevalent in HF cases and was independently associated with increased risk of new-onset HF. In addition CHIP correlates with HF risk factors ( smoking and hypertension) and biomarkers (N-terminal pro-B-type natriuretic peptide and mid-regional pro-A-type natriuretic peptide). |

| | | | |
|---|---|---|---|
| | | the prospective Prevention of Renal and Vascular End-stage Disease (PREVEND) cohort. | |
| Schuermans A, et al (2023) [91] | This study aims to investigate the association between birth weight and the development of CHIP in adulthood. | This study analysed whole exome data of 221 047 adults enrolled in the UK Biobank with self-reported birth weight. | CHIP was independently and additively associated with incident cardiovascular disease or death across birth weight groups, with highest absolute risks in those with CHIP plus high or low birth weight.<br>Higher birth weight is associated with increased risk of developing CHIP in midlife, especially *DNMT3A* CHIP |
| Dhindsa RS, et al (2023) [50] | This study aims to identify associations between rare protein-coding genetic variants and the abundances of 2,923 plasma proteins. | This study analysed whole exome sequencing data of 49,736 UK Biobank individuals. | The study identify distinct proteomic consequences of CH, including an association between *TET2*-CH and increased FLT3 levels. It suggested that the considerable role for rare variation in plasma protein abundance and the value of proteogenomics in therapeutic discovery |
| Lin AE, et al (2024) [92] | This study aimed to investigate the association between CHIP and the risk of developing atrial fibrillation (AF). | This study analysed whole exome sequencing data from 358 097 individuals of UK Biobank cohort to identify prevalence of CHIP for further analysis. | This study concluded that there is a modest association between CHIP, particularly *TET2* CHIP, and incident AF in the UK Biobank population. Validation in a AF mouse model with hematopoietic-specific Tet2 inactivation supported this finding, with altered calcium handling proposed as an arrhythmogenic mechanism dependent on NLRP3 inflammasome activation. |
| Schuermans A, et al (2024) [93] | This study aimed to test the association of CHIP with new-onset arrhythmias. | This study analyzed whole-exome sequencing data from 410,702 UK Biobank participants without a history of arrhythmias. | This study demonstrated that CHIP may represent a novel risk factor for incident arrhythmias, suggesting a potential target for prevention and therapeutic intervention. |

| | | | |
|---|---|---|---|
| Ezzat et al., (2025) [94] | To investigate the association of CHIP subtypes with CVD outcomes | This study analysed high coverage sequencing (median depth 4,580×) data from 6,677 older women (median age 80 years) participate in the Women's Health Initiative Long Life Study in between 2012 and 2013. | This study concluded, in an older female cohort, key CHIP subtypes (*TET2, ASXL1*, and *JAK2*) were associated with incident CVD, with gene-specific differences across outcomes. TET2 was linked to coronary disease (HR 1.35), whereas *JAK2* was associated with ischemic stroke and venous thromboembolism (VTE) (HR 2.49–2.71), highlighting subtype-specific cardiovascular risk in later life. |
| Shyr et al., (2026) [95] | To assess whether CHIP is associated with CVD risk in patients receiving cardiotoxic cancer therapy | This study analyzed whole-genome sequencing data from 250,038 participants in a cohort study conducted using BioVU, Vanderbilt University Medical Center's biorepository linking electronic health records from 2006 to 2025. | This study revealed, CHIP was associated with increased CVD risk in patients with solid tumors receiving cancer therapy. This finding suggests incorporating CHIP status may improve cardio-oncology treatment of cancer survivors. |
| Schuermans A, et al (2025) [96] | To test whether CHIP is associated with the development of myocarditis and pericarditis. | Participants with whole-exome sequencing data, no prevalent cardiovascular disease or hematological malignancy, and complete covariate information were included from the UK Biobank cohort, enrolled between 2006 and 2010. | In this study, CHIP was a strong risk factor for myocarditis and pericarditis among middle-aged adults. Targeting CHIP and its downstream pathways may represent a strategy for preventing or treating pericarditis and myocarditis. |

| | | | |
|---|---|---|---|
| Saadatagah et al. (2025) [97] | To investigate the association between CHIP and VTE in older adults. | This study analyzed whole-exome sequencing data from 3,980 participants (median age 75 years) in the ARIC cohort, who had no prior venous thromboembolism or hematologic malignancies. | This study concluded, over a median 7.1-year follow-up, CHIP carriers had a higher incidence of VTE compared with non-carriers (HR 1.49; 95% CI 1.02–2.17), with *TET2* mutations showing significant association with VTE risk (HR 2.25; 95% CI 1.27–4.00) |
| Zhang et al. (2025) [98] | To identify pathogenic genetic variants in familial VTE using whole-genome sequencing. | This study analyzed whole-genome sequencing data from 216 individuals across 35 Han Chinese venous thromboembolism (VTE) pedigrees and validated the findings in 99 high-heritability VTE cases using whole-exome sequencing. | These findings provide novel insights into the genetic architecture of VTE and highlight GP6, *TET2*, and *JAK2* as potential risk factors in East Asian populations, underscoring the clinical relevance of rare variants in VTE pathogenesis. |

**Table S2. CHIP driver genes linked to cardiovascular disease**

| Gene (position) | Description | Variant of mutation | Expression (cell specific) |
|---|---|---|---|
| TET2 (chr4q23) | **Function**: DNA demethylation<br>**Mutational effect**: Hypermethylation of DNA resulting from disruption of function of TET family dioxygenase<br>**Chemical properties**: A methylcytosine dioxygenase that catalyses the conversion of 5-hydroxymethylcytosine (5hmC) into 5-hydroxymethylcytosine. Act as key role player in activating DNA demethylation. Methylation at the C5 position of cytosine bases favoured an epigenetic modification, which controls transcriptional regulation in mammalian genome. | • Loss-of-function, deletion, frameshift, or nonsense of TET2 gene mutations<br><br>**Hotspot variants**<br>p.Arg550Ter | Highly expressed in peripheral blood mononuclear cells, especially in monocytes and macrophages<br>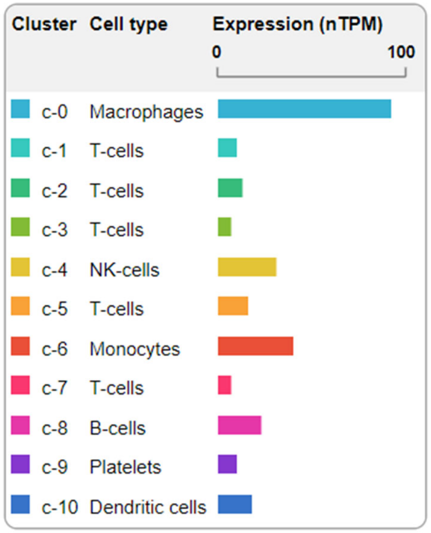 |

| | | | |
|---|---|---|---|
| DNMT3A (chr2p23) | **Function**: DNA methylation<br>**Mutation effect**: Hypomethylation of DNA methyltransferase enzyme accountable for the establishment of new DNA methylation during development and stem cell fate decisions.<br>**Chemical properties**: | Loss of function of DNMT3A gene, frameshift, or nonsense mutations<br><br>• cause loss-of-function<br>• enhance HSC self-renewal and promote the expression of multipotency genes while suppressing differentiation factor expression.<br>• Inducing pro-inflammatory T-cell polarization<br>• Activating inflammasome markers complex<br><br>**Hotspot variants**<br>p.Arg882His<br>p.Arg882Cys | Highly expressed in fetal tissues, skeletal muscle, heart, **peripheral blood mononuclear cell** and kidney. Acted as prognostic marker in cancer-related genes.<br><br>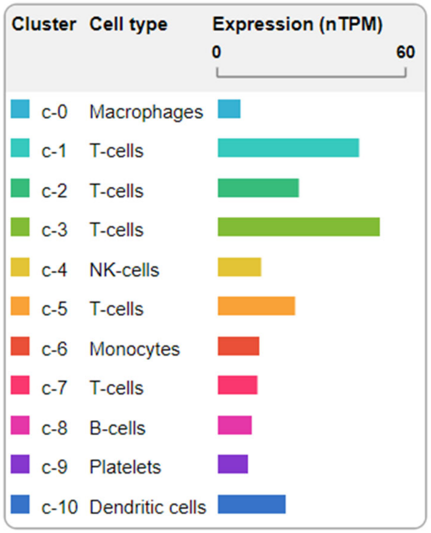 |

| | | | |
|---|---|---|---|
| ASXL1 (chr20q11) | **Function**: Chromatin modification<br>Mutational effect: Abnormal epigenetic regulation via interaction with polycomb recessive complex. Polycomb chromatin-binding protein that is involved in the transcriptional regulation | Recurrent truncating loss of fucntion exon 11 or 12 nonsense or frameshift mutations | Highly expressed in blood and immune cells, especially T-cells<br>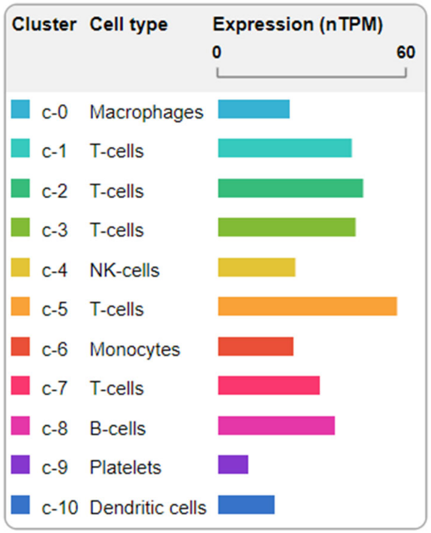 |
| JAK2 (chr9p24) | **Function**: Receptor tyrosine kinase<br>Mutational effect: Constitutive activation of JAK-STAT signalling pathway. Receptor tyrosine kinase involved in haematopoietic cytokine signalling and myelopoiesis | Single nucleotide variants conferring gain of function<br><br>**Hotspot variants**<br>p.Val617Phe | Highly expressed in immune cells, especially in macrophages and monocytes. |

| | | | |
|---|---|---|---|
| | | | 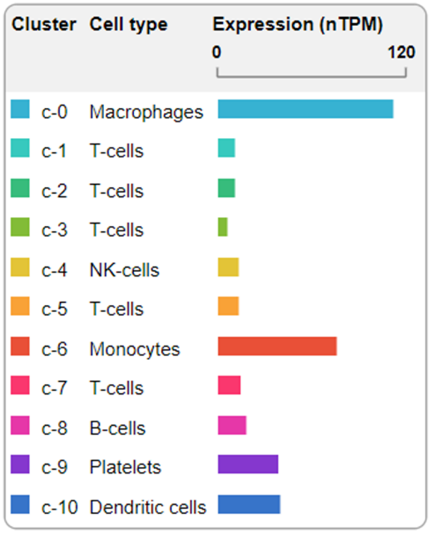 |
| TP53 (chr17) | **Function**: Tumor suppressor and regulate DNA damage response **Mutational effect**: Transcription factor regulating cell cycle arrest, apoptosis, senescence, DNA repair, and metabolism changes in response to diverse cellular stresses | Loss of function mutations often affecting the DNA, binding domain. Damage response pathway in regulatory feedback loop with the tumor suppressor p53. | Highly expressed in immune cells, especially monocytes, macrophages and B-cells |

| | | | |
|---|---|---|---|
| | | | 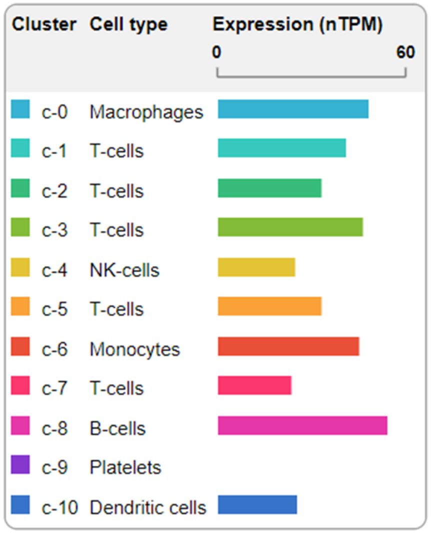 |
| PPM1D (chr 17 q23) | **Function:** regulate the cellular DNA damage response<br>**Mutational effect:** mutation induced by p53 and negatively regulates p38 MAPK DNA damage response pathway in regulatory feedback loop with the tumor suppressor p53 | Loss of function mutations are implicated in the dysregulation of cellular processes, particularly DNA damage response pathways. PPM1D, as a phosphatase, plays a role in regulating the cellular response to DNA damage. | Highly expressed in immune cells, especially monocytes, marcophages and B cells |

| | | | |
|---|---|---|---|
| | | | 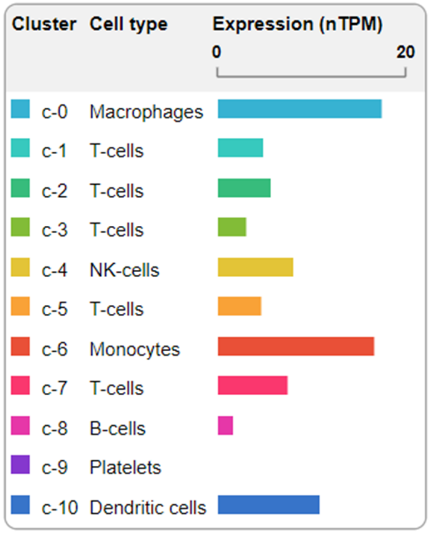 |



## References


1. Jaiswal, S., et al., *Age-related clonal hematopoiesis associated with adverse outcomes.* N Engl J Med, 2014. **371**(26): p. 2488-98.
2. Jaiswal, S., et al., *Clonal Hematopoiesis and Risk of Atherosclerotic Cardiovascular Disease.* N Engl J Med, 2017. **377**(2): p. 111-121.
3. Dorsheimer, L., et al., *Association of Mutations Contributing to Clonal Hematopoiesis With Prognosis in Chronic Ischemic Heart Failure.* JAMA Cardiol, 2019. **4**(1): p. 25-33.
4. Bick, A.G., et al., *Inherited causes of clonal haematopoiesis in 97,691 whole genomes.* Nature, 2020. **586**(7831): p. 763-768.

5. Bick, A.G., et al., *Genetic Interleukin 6 Signaling Deficiency Attenuates Cardiovascular Risk in Clonal Hematopoiesis.* Circulation, 2020. **141**(2): p. 124-131.

6. Busque, L., et al., *High-sensitivity C-reactive protein is associated with clonal hematopoiesis of indeterminate potential.* Blood Adv, 2020. **4**(11): p. 2430-2438.

7. Pascual-Figal, D.A., et al., *Clonal Hematopoiesis and Risk of Progression of Heart Failure With Reduced Left Ventricular Ejection Fraction.* J Am Coll Cardiol, 2021. **77**(14): p. 1747-1759.

8. Kar, S.P., et al., *Genome-wide analyses of 200,453 individuals yield new insights into the causes and consequences of clonal hematopoiesis.* Nature Genetics, 2022. **54**(8): p. 1155-1166.

9. Kessler, M.D., et al., *Common and rare variant associations with clonal haematopoiesis phenotypes.* Nature, 2022. **612**(7939): p. 301-309.

10. Nakao, T., et al., *Mendelian randomization supports bidirectional causality between telomere length and clonal hematopoiesis of indeterminate potential.* Science advances, 2022. **8**(14): p. eabl6579.

11. Yu, Z., et al., *Human Plasma Proteomic Profile of Clonal Hematopoiesis.* bioRxiv, 2024.

12. Stacey, S.N., et al., *Genetics and epidemiology of mutational barcode-defined clonal hematopoiesis.* Nat Genet, 2023. **55**(12): p. 2149-2159.

13. Yu, Z., et al., *Genetic modification of inflammation- and clonal hematopoiesis-associated cardiovascular risk.* J Clin Invest, 2023. **133**(18).

14. Mayerhofer, E., et al., *Prevalence and Therapeutic Implications of Clonal Hematopoiesis of Indeterminate Potential in Young Patients With Stroke.* Stroke, 2023. **54**(4): p. 938-946.

15. Gumuser, E.D., et al., *Clonal Hematopoiesis of Indeterminate Potential Predicts Adverse Outcomes in Patients With Atherosclerotic Cardiovascular Disease.* J Am Coll Cardiol, 2023. **81**(20): p. 1996-2009.

16. Shi, C., et al., *Clonal haematopoiesis of indeterminate potential: associations with heart failure incidence, clinical parameters and biomarkers.* Eur J Heart Fail, 2023. **25**(1): p. 4-13.

17. Schuermans, A., et al., *Birth Weight Is Associated With Clonal Hematopoiesis of Indeterminate Potential and Cardiovascular Outcomes in Adulthood.* J Am Heart Assoc, 2023. **12**(13): p. e030220.

18. Dhindsa, R.S., et al., *Rare variant associations with plasma protein levels in the UK Biobank.* Nature, 2023. **622**(7982): p. 339-347.

19. Lin, A.E., et al., *Clonal Hematopoiesis of Indeterminate Potential With Loss of Tet2 Enhances Risk for Atrial Fibrillation Through Nlrp3 Inflammasome Activation.* Circulation, 2024. **149** (18): p. 1419-1434

20. Schuermans, A., et al., *Clonal haematopoiesis of indeterminate potential predicts incident cardiac arrhythmias.Eur Heart J. 2024.* ***45****(10):p. 791-805.*

21. Ezzat D, et al., *Clonal Hematopoiesis and Cardiovascular Outcomes in Older Women. J Am Coll Cardiol. 2025;****86****(15):1093-1106.*

22. Shyr D, et al*., Clonal Hematopoiesis and Cardiovascular Disease Risk After Cancer Therapy in Patients With Solid Tumors. JAMA Oncol. Published online January 08, 2026. doi:10.1001/jamaoncol.2025.5785*

23. Schuermans A, et al., *Clonal Hematopoiesis and Risk of New-Onset Myocarditis and Pericarditis. JAMA Cardiol. 2025;**10**(11):1147–1156.*

24. Saadatagah S, et al., *Clonal hematopoiesis of indeterminate potential and incidence of venous thromboembolism in older adults. J Thromb Haemost. 2025;**23**(7):2235-2241.*

25. Zhang Z, et al., *Whole genome sequencing identifies pathogenic genetic variants in Han Chinese patients with familial venous thromboembolism. Commun Biol. 2025;**8**(1):604.*
